# Lack of Expression of Gp-130 Makes Pancreatic Beta Cell Lines Unresponsive to the IL-6 Family of Cytokines

**DOI:** 10.1155/EDR.2000.239

**Published:** 2000

**Authors:** Gaetano Naselli, Henry J. Deaizpurua, Helen E. Thomas, Anne M. Johnston, Thomas W. H. Kay

**Affiliations:** 1 Autoimmunity and Transplantation Division Walter and Eliza Hall Institute of Medical Research Royal Parade, Parkville Melbourne Australia; 2 The Walter and Eliza Hall Institute P.O Royal Melbourne Hospital Victoria 3050 Australia

## Abstract

Cytokine receptors from the IL-6 receptor family are comprised of ligand specific α chains and a common signalling chain, gp-130, which is also required for high affinity binding. A cDNA library generated from the β-TC3 SV40 T-antigen transformed insulinoma cell line was screened for members of this receptor family potentially relevant to both beta cell development and autoimmunity. Degenerate oligonucleotide primers to a consensus region of these receptors were used and the IL-11 receptor (α chain was identified. Despite confirmation of IL-11 receptor mRNA expression, iodinated bioactive IL-11 did not bind specifically to β-TC3 cells and gp-130-dependent cytokines did not elicit signalling events in beta cell lines. This was explained by absence of gp-130 protein or mRNA in the beta cell lines tested and in primary islets. We conclude from these resuits that the previously recognised effects of IL-6 family member cytokines on pancreatic islets must be indirect *via* other non-beta cells within the islet, rather than due to direct effects on beta cells themselves.


*Int. Jnl. Experimental Diab. Res.*, Vol. 1, pp. 239–248
Reprints available directly from the publisher
Photocopying permitted by license only

© 2001 OPA (Overseas Publishers Association) N.V.
Published by license under
the Harwood Academic Publishers imprint,
part of The Gordon and Breach Publishing Group.
Printed in Malaysia.

### Lack of Expression of Gp-130 Makes Pancreatic Beta Cell Lines Unresponsive to the IL-6 Family of Cytokines

GAETANO NASELLI, HENRY J. DEAIZPURUA, HELEN E. THOMAS, ANNE M. JOHNSTON and THOMAS W. H. KAY*

*Autoimmunity and Transplantation Division, Walter and Eliza Hall Institute of Medical Research, Royal Parade, Parkville, Melbourne, Australia*

*(Received in final form 11 May 000)*

**Cytokine receptors from the IL-6 receptor family are comprised of ligand specific α chains and a common signalling chain, gp-130, which is also required for high affinity binding. A cDNA library generated from the β-TC3 SV40 T-antigen transformed insulinoma cell line was screened for members of this receptor family potentially relevant to both beta cell development and autoimmunity. Degenerate oligonucleotide primers to a consensus region of these receptors were used and the IL-11 receptor α chain was identified. Despite confirmation of IL-11 receptor mRNA expression, iodinated bioactive IL-11 did not bind specifically to β-TC3 cells and gp-130-dependent cytokines did not elicit signalling events in beta cell lines. This was explained by absence of gp-130 protein or mRNA in the beta cell lines tested and in primary islets. We conclude from these results that the previously recognised effects of IL-6 family member cytokines on pancreatic islets must be indirect *via* other non-beta cells within the islet, rather than due to direct effects on beta cells themselves.**

*Keywords*: Beta cell; Cytokine receptors; Insulinoma cell lines; Signal transduction

### INTRODUCTION

The IL-6 receptor is a member of a cytokine receptor family which includes IL-11, LIF, oncostatin M (OSM), ciliary neurotrophic factor (CNTF) and cardiotrophin-1 receptors. These receptors utilize a common receptor chain, gp-130, for signal transduction. Although the primary effects of IL-6 and LIF have been described on cells within the haematopoeitic lineage [1] they also act to promote gene expression, growth and differentiation of non-haematopoietic cells [2–4] (reviewed in Nicola, 1998). Whether or not cytokines of this family have an effect on pancreatic beta cell development is unknown, however regenerative effects on endocrine cells have been described in IL-6 transgenic mice. [5] Other members of the IL-6 receptor family have also been shown to affect development of a variety of cell types. IL-11, for

*Address for correspondence: The Walter and Eliza Hall Institute, P.O Royal Melbourne Hospital, 3050 Victoria, Australia. Tel.: 61-3-9345 2457, Fax: 61-3-9347 0852, e-mail: kay@wehi.edu.au example, is known to stimulate haematopoiesis, B lymphocyte function,[6,7] small intestinal cell recovery following cytoablative therapy[8] and neuronal differentiation of hippocampal progenitor cells.[9] Another IL-6 family member, CNTF, has been shown to support the survival of parasympathetic neurones from the chick ciliary ganglion,[10] while enhancing the survival of sensory neurones,[11] motor neurones[12] and hippocampal neurones.[13]

Type 1 (insulin-dependent) diabetes is a T cell mediated autoimmune disease in which the pancreatic beta cells are selectively destroyed. A role for the IL-6 family of cytokines in autoimmune diabetes has been postulated. IL-6 is produced within islets, possibly by endocrine as well as inflammatory cells, and administration of neutralizing antibody to IL-6 decreased progression of diabetes in the non-obese diabetic (NOD) mouse.[14] IL-6 was shown to have direct effects on insulin secretion by rat islets *in vitro*,[15,16] and to enhance the inhibitory effects of IL-1 on rat islets. Recently, CNTF was also shown to enhance the destructive effects of IL-1 on rat islets by enhancing IL-1-mediated nitric oxide production by beta cells.[17] CNTF mRNA was also detected in rat islet cells.

Using a degenerate oligonucleotide screening strategy to identify known or novel members of the IL-6 cytokine receptor family involved in beta cell function, we have found expression of the IL-11 receptor on a beta cell line. However, when the functional effects of the IL-11 receptor and other members of this family were examined on beta cell lines, we were unable to detect an effect. We demonstrate that this is due to absence of the signalling component of the receptor, gp-130, on both beta cell lines and primary murine islets.

### MATERIALS AND METHODS

### Cell Culture and Cytokines

Mouse insulinoma ($\beta$-TC3, NIT) cells were continuous clonal cell lines obtained from the American Type Tissue Culture (ATCC, Rockville, MD) derived from transgenic mice expressing the SV-40 large T-antigen under the control of the rat insulin promoter.[18,19] $\beta$-TC3 cells were derived from transgenic C57BL/6JxDBA/2J mice whereas NIT cells were derived from transgenic non-obese diabetic (NOD) mice. MUR21 and M127 myeloid leukaemia cells were the gift of Dr. Doug Hilton (Walter and Eliza Hall Institute, Melbourne, Australia). These cells were maintained at 37°C in 10% $CO_2$ in Dulbecco's minimal essential medium (DMEM) supplemented with 10% foetal bovine serum (FBS), glucose (16.5 mM), non-essential amino acids and antibiotics. NIH 3T3 cells were maintained at 37°C in 10% $CO_2$ in DMEM with 5% FBS and antibiotics. Pancreatic islets isolated from both NOD and C57BL/6xSJL mice were purified and maintained in short term culture as described.[20] Mouse embryos were dissected, the pancreata removed and total RNA isolated using RNAzol (Tel-Test Inc., Friendswood, TX) according to the manufacturer's instructions. Murine IL-3 and IL-11 were used as culture supernatants from WEHI 3BD$^-$. Recombinant murine IL-6 and LIF were from Dr. Richard Simpson (Ludwig Institute, Melbourne, Australia) and used at 10 ng/ml and 500 U/ml respectively. Insulin was measured by radioimmunoassay (Phadeseph, Pharmacia, Uppsala, Sweden).

### Antibodies

Anti-gp-130 antibody (M-20, Santa Cruz Biotechnology, Santa Cruz, CA) was an affinity-purified rabbit polyclonal antibody raised against amino acids 895–914 corresponding to the carboxy-terminus of murine gp-130. Monoclonal anti-ISGF3 91/84 (Transduction Laboratories, Lexington, KY) and polyclonal anti-STAT3 (Santa Cruz Biotechnology, Santa Cruz, CA) were used to identify complexes in electrophoretic mobility shift assays (EMSAs). Control antibodies were derived from pre-immune rabbits or mice as required.

### Library Screening

Approximately $1 \times 10^6$ plaques from a $\beta$-TC3 $\lambda$-ZAP cDNA library were screened by colony lift procedures as described.[21] Briefly, infected bacteria (XL1-Blue) were grown on 150 mm plates and plaques transferred to duplicate 150 mm membranes (Colony/Plaque Screen, NEN Research products, Boston, MA). Denatured, fixed, bacterial filters were rinsed with 2XSSC (150 mM sodium chloride, 15 mM sodium citrate dihydrate), prior to pre-hybridisation overnight in hybridisation buffer (6X SSC, 2 mg/ml BSA, 2 mg/ml Ficoll, 100 µM ATP, 10 µg/ml tRNA, 2 mg/ml polyvinylpyrrolidone, 2 mg/ml salmon sperm DNA, 200 µg/ml sodium azide and 0.1%(w/v) SDS) at 42°C. Degenerate oligonucleotide primers [300 ng-WSAWS; 5′-TGGAG(T/C)GC(A/C/G/T)-TGGAG(T/C)-3′, WSEWS; 5′-TGGAG(T/C) GA(A/G)TGGAG(T/C)-3′, WSDWS; 5′-TGGAG-(T/C)GA(C/T)TGGAG(T/C)] were phosphorylated by end labelling with T4 polynucleotide kinase using 160 µCi [$\gamma$-$^{32}$P] ATP (Promega, Madison, Wi, USA). A pre-packed gel filtration column (NAP-5, Pharmacia, Uppsala, Sweden) was employed to separate unincorporated ATP from labelled probes and filters were hybridised overnight at 42°C in hybridisation buffer. Filters were rinsed twice at room temperature in 6XSSC, 0.1% SDS, washed twice in 6XSSC, 0.1% SDS at 48°C and rinsed twice with 2XSSC at room temperature, prior to drying by brief blotting then mounting for autoradiography at −70°C using Kodak X-Omat-AR film and intensifying screens, for 7 days. Positive plaques on duplicate filters were selected, eluted in 1 ml 100 mM NaCl, 10 mM $MgCl_2$, 10 mM Tris-HCl, pH 7.4 with 0.5% (w/v) gelatin and 0.5% (v/v) chloroform and stored at 4°C. XL1-Blue cells were subsequently infected with the eluate from the primary plugs and replated for a secondary screen. This procedure was repeated a third time to obtain pure plaques.

### Identification of Plaques

Positive plaques were screened for inserts by PCR using primer combinations generated to 5′- and 3′ flanking regions of pBK-CMV phagemid vector (Stratagene Cloning Systems, La Jolla, CA) as follows:

5′-GATTACGCCAAGCTCGAAATTAACC-3′

(HN1-forward primer)

5′-TACGACTCACTATAGGGCGAATTGG-3′

(HN2-reverse primer)

PCR conditions were 94°C/3′ + 35cycles (94°C/1′, 50°C/1′, 72°C/3′) + 72°C/5′ + 4°C in 20 µl final volume. The products were resolved on 1.5% agarose gels. Clones containing insert were prepared for sequencing by dideoxy terminator sequencing using a 320A sequenator (Applied Biosystems, Foster City, CA). The BLAST algorithm[22] was used to search databases for protein and DNA homologies.

### Northern Analyses

Total or poly $(A)^+$-selected RNA transcripts (purified using PolyATract mRNA isolation system III, Promega, Madison, WI) were examined by Northern analyses as described.[23] Briefly, RNA was electrophoresed on a 1% (v/v) formaldehyde agarose gel and transferred to Genescreen Plus (Dupont, NEN) nitrocellulose membrane in 10X SSC. Blots were pre-hybridised in ExpressHyb (Clontech Laboratories, Palo Alto, CA), for 30 min at 68°C. cDNA probes were labelled with $\alpha$-[$^{32}$P] dATP (Dupont, NEN, 3000Ci/mmol) by random priming to a specific activity of $1 \times 10^8$ cpm/µg, added to filters in prehybridisation solution, and incubated for 1 h at 68°C. The membrane was washed at RT in 2x SSC containing 0.05% (w/v) SDS for 1 h, at 65°C in 1x SSC containing 0.1% (w/v) SDS for 1h, and exposed to autoradiography at −70°C.

### Immunoblotting

Cells were lysed in lysis buffer A (10 mM CHAPS, 50 mM Hepes, pH 7.6, 150 mM NaCl, 3 mM phenylmethylsulphonyl fluoride (PMSF), 20 μg/ml leupeptin, 20 μg/ml aprotinin (Sigma, St Louis, MO)) and protein concentration was estimated using commercially prepared reagent (Bio-Rad, Hercules, CA). Proteins from cell lysates (20 μg/lane) were resolved by SDS-PAGE, electroblotted onto nitrocellulose membranes (Schleicher and Schuell Germany), and blocked for 16 h in 5% skim milk in phosphate buffered saline (PBS).

Membranes were incubated with primary antibody at 0.5 μg/ml (final) for 2 h at 25°C, followed by four washes in PBS and incubation with anti-rabbit horseradish peroxidase-labelled secondary antibody (Silenus, Australia) diluted 1:1000 for 1 h at 25°C. Following five washes in PBS, blots were developed with ECL reagent (Amersham International, Amersham, UK) and bands detected by autoradiography.

### Binding Studies with IL-11

Binding studies were performed essentially as described by Hilton and Nicola.[24] Briefly, $10^6$ cells in 40 μl RPMI-1640 or DME medium containing 20 mM Hepes, pH7.4 and 10% (v/v) FCS were incubated overnight on ice with $1 \times 10^5$ cpm $^{125}$I-labelled IL-11 with or without a 100-fold excess of unlabelled IL-11. Cell associated and free $^{125}$I-labelled IL-11 were separated by rapid centrifugation through 180 μl FCS and quantitated in a $\gamma$-counter.

### Electrophoretic Mobility Shift Assay (EMSA)

Nuclear extracts were prepared as described.[25] Briefly, cells were lysed in lysis buffer B (10 mM Hepes pH 7.9, 10 mM KCl, 1.5 mM $MgCl_2$, 0.5 mM PMSF, 10 μg/ml leupeptin, 0.5 mM DTT). The lysate was vortexed with 25 μl 10% NP40 and centrifuged. The pellet was resuspended in 420 mM NaCl, 20 mM Hepes pH 7.9, 1.5 mM $MgCl_2$, 0.2 mM EDTA, 10% glycerol, 0.5 mM PMSF, 10 μg/ml leupeptin and 0.5 mM DTT and centrifuged at 12,000 rpm at 4°C for 5 min. EMSAs were carried out using oligonucleotides labelled with $\alpha$-[$^{32}$P] dATP by end-labelling as described previously. The sequence of the *c*-sis-inducible element probe (M67) used was 5′-GATCTAGGGATTTCCGG-GAAATGAAGCT-3′.[26] EMSAs containing 2 μl of nuclear extract were incubated on ice in reaction buffer (13.3 mM Hepes pH 7.6, 0.7 mM EDTA. 3.3 mM $MgCl_2$, 34 mM KCl, 1 mM DTT, 10% glycerol and 0.5 μg poly (dI-dC)). Reactions proceeded for 15 min followed by the addition of $^{32}$P-labelled oligonucleotide ($\sim$15,000 dpm). Competitor cDNA was added at 100x molar excess.

When antibodies specific for transcription factors were employed in EMSAs, antibody was reacted with extracts for 1 h on ice prior to the addition of labelled oligonucleotide. After 15 min, the DNA-protein complexes were analysed by electrophoresis through a 5% non-denaturing polyacrylamide gel in 0.25x TBE (TBE is 50 mM Tris, 50 mM boric acid and 1 mM EDTA). Gels were subsequently dried and exposed to x-ray films at −70°C.

### RESULTS

### Identification of Cytokine Receptors

We screened a pancreatic beta cell line, $\beta$-TC3, to examine cytokine receptors which may be important during endocrine cell development. Degenerate oligonucleotides corresponding to the cytokine receptor homology domain: Trp-Ser-Ala-Trp-Ser (WSAWS) were employed as a probe to screen $\sim 10^6$ plaques from a $\beta$-TC3 $\lambda$-ZAP cDNA library. We identified two independent cDNAs encoding the murine IL-11 receptor alpha chain. The sequences obtained corresponded to nt19–170 of the full-length, murine IL-11 receptor alpha chain. This was of immediate interest to us because of previous data implicating cytokines related to IL-11 in the pathogenesis of diabetes.

### Lack of IL-11 Binding to, and Effects on, $\beta$-TC3 Cells

To determine whether IL-11 could directly bind to the high affinity IL-11 receptor, $^{125}$I-labelled IL-11 was reacted with $\beta$-TC3 cells or the IL-11 receptor/gp-130 deficient myeloid leukaemia cell line MUR21, or M1 cells (which express gp-130) stably transfected with IL-11 receptor (M127). Radioiodinated IL-11 bound specifically to the high affinity receptor-containing M127 cells but not to the receptor-deficient MUR21 cells or to $\beta$-TC3 cells (Fig. 1).

IL-11 and other members of this family of cytokines signal *via* activation of the transcription factors STAT1 and STAT3. Phosphorylated STATS 1 and 3 homo- and hetero-dimerize and are then able to bind to a modified serum

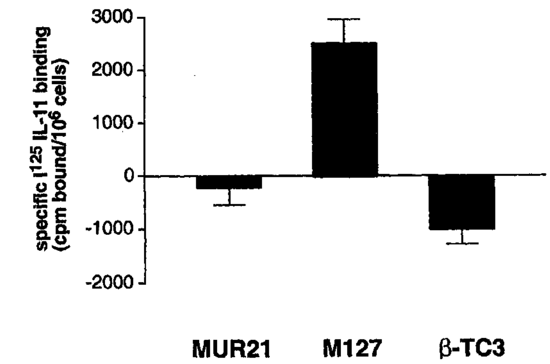

FIGURE 1 *Radioligand binding*. Specific binding of radioiodinated IL-11 to $\beta$-TC3 cells, IL-11 receptor positive M127 cells and IL-11 receptor deficient MUR21 cells. Results are the mean +2 S.D. of two experiments each performed in triplicate.

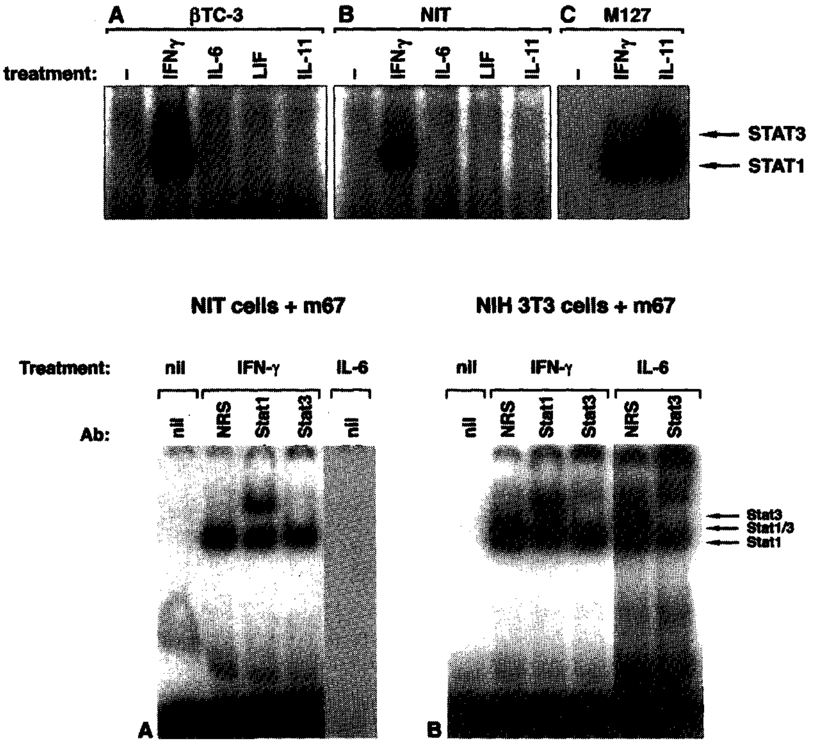

FIGURE 2 *Signalling through gp-130*. Electrophoretic mobility shift assays (EMSAs) showing binding to oligonucleotide M67 of nuclear proteins in extracts from $\beta$-TC3 (*A*), and NIT (*B*) cells after treatment with IFN-$\gamma$, IL-6, LIF, IL-11. M127 cells were treated with IFN-$\gamma$ or IL-11 (*C*). Shifted complexes representing STAT1 and STAT1/3 are indicated. *D*, EMSA of NIT cells (*a*) and NIH 3T3 cells (*b*) after 30 min treatment with IFN-$\gamma$ or IL-6. Extracts were pre-incubated with antiserum (Ab) to STAT1, STAT3 or pre-immune rabbit serum (NRS). Complexes containing either STAT1, STAT3 or both are "super-shifted" or decreased in intensity following incubation with specific antibodies. IL-6 treated NIT extracts were not incubated with anti-sera because no IL-6 induced complex was observed.

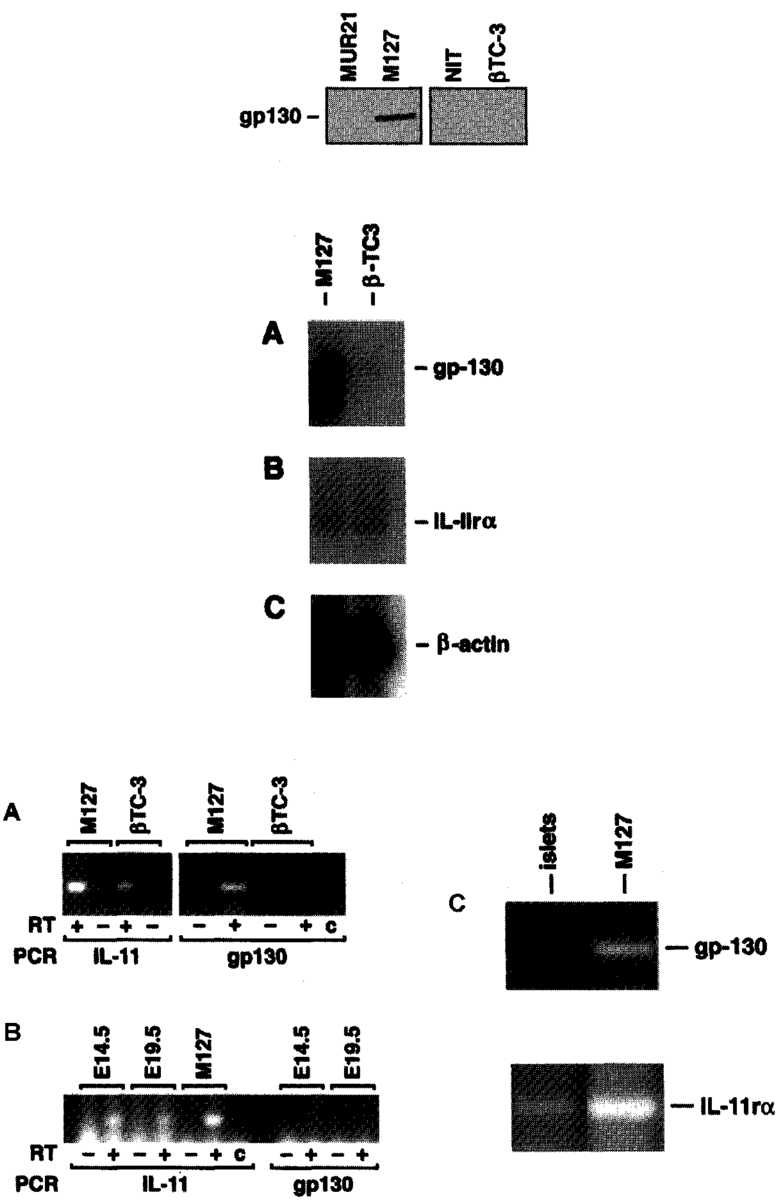

FIGURE 3 *Expression of IL-11 and gp-130 receptors*. Expression of gp-130 protein on beta cells. (A) Western blot of cell lysates from β-TC3, NIT, or gp-130 containing haematopoietic cells, probed with anti-gp-130 polyclonal antibody. (B) Autoradiograph of mRNA from β-TC3 cells and M127 cells probed with radiolabelled cDNA for gp-130 (*A*), IL-11 receptor α-chain (*B*) or mouse β-actin (*C*). (C) RT-PCR analyses of IL-11 receptor and gp-130 expression. RT-PCR of mRNA from M127 and β-TC3 cells (*A*), embryonic mouse pancreas (*B*) or isolated mouse islets (*C*), amplified with primers coding for cDNAs from murine IL-11 receptor α-chain or murine gp-130. Reactions performed in the absence of DNA template are designated c.

responsive element termed M67. While β-TC3 cells were capable of responding to IFN-γ with an appropriate STAT1 product in an electrophoretic mobility shift assay (EMSA), we could not detect STAT1/3 heterodimer or STAT3 homodimers in response to IL-11, IL-6 or LIF (Fig. 2A). To confirm that these observations were not restricted to β-TC3 cells we examined

NIT cells derived from SV-40 T-antigen transgenic non-obese diabetic (NOD) mice. While NIT cells treated with IFN-$\gamma$ produced a STAT1 band shift, IL-11, IL-6 and LIF did not (Fig. 2B). In contrast, M1 myeloid leukaemia cells stably transfected with the IL-11 receptor (M127 cells) had bands consistent with STAT1 and STAT1/3 complexes after cytokine treatment (Fig. 2C). Anti-STAT1 and anti-STAT3 antibodies were used to identify STAT complexes in supershifts of lysates of NIT cells or NIH 3T3 cells after treatment with IFN-$\gamma$ or IL-6. Although both NIT and NIH 3T3 cells contained phosphorylated STAT1 homodimers that could be detected by supershift with anti-STAT1 antibodies after IFN-$\gamma$ treatment, only NIH 3T3 cells could respond to IL-6 treatment with subsequent phosphorylation of STAT3 and supershift by anti-STAT3 antibodies (Fig. 2D). Consistent with lack of cytokine receptor activation, no effects of IL-11, IL-6 or LIF were observed on insulin secretion or growth rates of $\beta$-TC3 cells (data not shown). It was not clear that these assays could rule out actions of these cytokines that may be relevant to the pathogenesis of autoimmune diabetes (*e.g.*, stimulation of chemokine expression, increased susceptibility to other inflammatory effector molecules *etc.*). Because of our interest in evidence linking these cytokines to diabetes we decided to look further into why no effects were detected.

### Failure to Detect gp-130 Expression in Beta Cells

To determine whether the failure of beta cells to respond to cytokines was caused by a deficiency in the adapter and signalling molecule gp-130, protein and mRNA expression was examined. Extracts from the beta cell lines ($\beta$-TC3 and NIT) and from positive (M127) and negative (MUR21) control cell lines were immunoblotted with gp-130 specific antibodies. A band of appropriate size was detected with anti-gp-130 antibodies in detergent extracts from M127 cells but not in extracts from $\beta$-TC3, NIT or receptor deficient MUR21 cells (Fig. 3A). IL-11 $\alpha$ chain mRNA was expressed in both M127 cells and $\beta$-TC3 cells but no transcript for gp-130 was detected in mRNA from $\beta$-TC3 cells as shown by Northern blot (Fig. 3B) or RT-PCR (Fig. 3C). Similarly no gp-130 transcript was found in primary islets by RT-PCR and no gp-130 protein was detected in islets by immunoblotting (not shown). Gp-130 cDNA was not detected in the developing pancreas at any stage of development examined (Fig. 3C). We have been unable to find gp-130 in the RNA samples tested which have come from a limited range of insulinoma cell lines and islets isolated from more than one mouse strain, indicating that mouse beta cells in general are unlikely to be able to respond *via* gp-130.

### DISCUSSION

The role of cytokines in endocrine cell function is unclear, however several reports have suggested the potential involvement of the IL-6 family of cytokines in $\beta$-cell development. Indeed IL-6 and CNTF have been shown to be secreted by mature pancreatic $\beta$-cells, raising the possibility that locally produced cytokines could provide important paracrine or autocrine signals to endocrine tissue.[27–29] We have employed a cDNA library screening strategy, using oligonucleotides to the conserved WSA/D/EWS motif of the cytokine receptor family to identify cytokine receptors that may have a role in endocrine cell function.

We identified mRNA for the $\alpha$-chain of the IL-11 receptor. The IL-11 receptor $\alpha$-chain could be detected in both transformed mouse cell lines and freshly isolated mouse islets (data not shown). These observations would suggest a potential role for this recently described member of the IL-6 cytokine receptor family in $\beta$-cells. However, we consistently failed to observe a response to IL-11 by $\beta$-cell lines in culture. In addition we could not detect specific binding of radioiodinated IL-11 to $\beta$-cells when compared to a control haematopoietic cell line. These observations suggested that a component of the receptor complex might not be functional.

The overlapping biological and biochemical activities of the IL-6 family of cytokines have been attributed to the sharing of an affinity-converting signal-transducing molecule, gp-130 by distinct cytokine receptor subunits.[30] The mature gp-130 molecule is a transmembrane glycoprotein that incorporates the conserved cytokine receptor homology domain (four *n*-terminal cysteines and a *c*-terminal WSXWS motif) on the second and third extracellular fibronectin-like repeats. Ligand binding results in gp-130 phosphorylation on tyrosine and the subsequent transmission of an intracellular signal. The IL-11 receptor is critically dependent on gp-130 for both high affinity ligand binding and subsequent signal transduction.[21] We could not detect murine gp-130 mRNA or protein in $\beta$-cell lines, despite being able to detect gp-130 in control cells. These observations were supported by the failure of IL-11, IL-6 and LIF to induce phosphorylation of STAT signalling molecules in cultured $\beta$-cells, a pathway dependent on gp-130.[31,32] In contrast, these cells could readily respond through STATs to IFN-$\gamma$. These data show that transformed $\beta$-cells are deficient in gp-130. The observation that whole islets did not contain detectable levels of gp-130 suggests that $\alpha$ and $\delta$-cells, which together with $\beta$-cells comprise the majority of the cells in the islet, may also be deficient in gp-130. Indeed RT-PCR analyses of transformed murine $\alpha$-cells failed to show transcripts for gp-130 (data not shown).

While we are reporting an essentially negative study, the results are important in interpreting the literature on the interactions between this family of cytokines and pancreatic beta cells. Deficiency of gp-130 on both mature and developing endocrine cells suggests that this family of cytokines has no role in endocrine cell development. However, it is possible that cytokine mediated developmental effects could occur between E9 and E14 when the pancreatic primordia first appears. The IL-6 family of cytokines has been implicated in the pathogenesis of autoimmune diabetes. IL-6 has been reported to affect glucose mediated insulin release[33] and islet insulin content and oxidation[34], although these effects were dependent on co-incubation with IL-1. A recent report has stated that CNTF and IL-6 are able to potentiate the IL-1-induced inhibition of insulin secretion by rat islets. Our data suggests that this can only be due to indirect effects of these cytokines on other cells within the islet such as resident leukocytes.[17] These cells might then release other factors capable of directly interacting with beta cells such as IL-1. In a transgenic model, expression of IL-6 under the control of the insulin promoter was reported to result in a complex, localised inflammatory reaction with islet hyperplasia.[5] In contrast, it has been reported that IL-6 does not affect transformed rat $\beta$-cell proliferation or hormone secretion and is not synergistic with IFN-$\gamma$ or TNF-$\beta$.[35] IL-6 has also been reported not to affect cAMP levels in foetal rat pancreatic islets.[36]

Although a link between the ability of $\beta$-cells to produce IL-6 and $\beta$-cell destruction has been suggested, no convincing evidence directly demonstrates a specific response to IL-6 family cytokines by $\beta$-cells. Our own studies on the effects of cytokines on the regulation of glutamate decarboxylase synthesis by isolated rat islets have shown that while IL-1$\beta$ and IFN-$\gamma$ had significant inhibitory effects, IL-6 and LIF had no effect.[37] In light of the current data showing the absence of gp-130 expression it is not surprising that no effect was seen.

In conclusion, we have demonstrated the presence of $\alpha$-chains for the IL-11 receptor on cultured $\beta$-cells and isolated rodent islets. This cytokine receptor, however, is functionally inactive because the signal transducing co-receptor molecule gp-130 is not expressed on these tissues. In addition, both LIF and IL-6 failed to induce either biological or signalling events on cultured $\beta$-cells. These observations have implications for the interpretation of studies using these cytokines to understand the destructive or inflammatory mechanisms involved in the pathogenesis of diabetes. There is a long list of potential effector mechanisms of beta cell destruction in autoimmune diabetes. These include membrane bound and soluble products of T cells and macrophages. Our strategy is to directly test each of these in turn. The fact that gp-130 is not expressed on mouse beta cells eliminates this family of cytokines as direct mediators of beta cell destruction but does not rule out possible indirect effects.

### *Acknowledgements*

These studies were supported by the Juvenile Diabetes Foundation International through the Susan and Angelo Alberti Diabetes Interdisciplinary Research Program, the National Health and Medical Research Council of Australia (Regkey 973002) and by AMKAID Pty. Ltd. TWHK holds a Career Development Award of the JDF.

### *References*

[1] Nicola, N. A. and Hilton, D. J. (1998). General classes and functions of four-helix bundle cytokines, *Adv. Prot. Chem.*, **52**, 1–65.
[2] Van Damme, J., Opdenakker, G., Simpson, R. J., Rubira, M. R., Cayphas, S., Vink, A., Billiau, A. and Van Snick, J. (1987). Identification of the human 26-kD protein, interferon beta 2 (IFN-beta 2), as a B cell hybridoma/plasmacytoma growth factor induced by interleukin 1 and tumor necrosis factor, *J. Exp. Med.*, **165**, 914–9.
[3] Horii, Y., Muraguchi, A., Iwano, M., Matsuda, T., Hirayama, T., Yamada, H., Fujii, Y., Dohi, K., Ishikawa, H., Ohmoto, Y. *et al.* (1989). Involvement of IL-6 in mesangial proliferative glomerulonephritis, *J. Immunol.*, **143**, 3949–55.
[4] Satoh, T., Nakamura, S., Taga, T., Matsuda, T., Hirano, T., Kishimoto, T. and Kaziro, Y. (1988). Induction of neuronal differentiation in PC12 cells by B-cell stimulatory factor 2/interleukin 6, *Mol. Cell. Biol.*, **8**, 3546–9.
[5] Campbell, I. L., Hobbs, M. V., Dockter, J., Oldstone, M. B. and Allison, J. (1994). Islet inflammation and hyperplasia induced by the pancreatic islet-specific overexpression of interleukin-6 in transgenic mice, *Am. J. Pathol.*, **145**, 157–66.
[6] Yin, T. G., Schendel, P. and Yang, Y. C. (1992). Enhancement of *in vitro* and *in vivo* antigen-specific antibody responses by interleukin 11, *J. Exp. Med.*, **175**, 211–6.
[7] Anderson, K. C., Morimoto, C., Paul, S. R., Chauhan, D., Williams, D., Cochran, M. and Barut, B. A. (1992). Interleukin-11 promotes accessory cell-dependent B-cell differentiation in humans, *Blood*, **80**, 2797–804.
[8] Du, X. X. and Williams, D. A. (1994). Interleukin-11: a multifunctional growth factor derived from the hematopoietic microenvironment, *Blood*, **83**, 2023–30.
[9] Mehler, M. F., Rozental, R., Dougherty, M., Spray, D. C. and Kessler, J. A. (1993). Cytokine regulation of neuronal differentiation of hippocampal progenitor cells, *Nature*, **362**, 62–5.
[10] Stockli, K. A., Lottspeich, F., Sendtner, M., Masiakowski, P., Carroll, P., Gotz, R., Lindholm, D. and Thoenen, H. (1989). Molecular cloning, expression and regional distribution of rat ciliary neurotrophic factor, *Nature*, **342**, 920–3.
[11] Skaper, S. D. and Varon, S. (1986). Age-dependent control of dorsal root ganglion neuron survival by macromolecular and low-molecular-weight trophic agents and substratum-bound laminins, *Brain Res.*, **389**, 39–46.
[12] Oppenheim, R. W., Prevette, D., Yin, Q. W., Collins, F. and MacDonald, J. (1991). Control of embryonic motoneuron survival *in vivo* by ciliary neurotrophic factor, *Science*, **251**, 1616–8.
[13] Ip, N. Y., Nye, S. H., Boulton, T. G., Davis, S., Taga, T., Li, Y., Birren, S. J., Yasukawa, K., Kishimoto, T., Anderson, D. J. *et al.* (1992). CNTF and LIF act on neuronal cells *via* shared signaling pathways that involve the IL-6 signal transducing receptor component gp130, *Cell*, **69**, 1121–32.
[14] Campbell, I. L., Kay, T. W., Oxbrow, L. and Harrison, L. C. (1991). Essential role for interferon-gamma and interleukin-6 in autoimmune insulin-dependent diabetes in NOD/Wehi mice, *J. Clin. Invest.*, **87**, 739–42.
[15] Buschard, K., Aaen, K., Horn, T., Van Damme, J. and Bendtzen, K. (1990). Interleukin 6: a functional and structural *in vitro* modulator of beta-cells from islets of Langerhans, *Autoimmunity*, **5**, 185–94.
[16] DiCosmo, B. F., Picarella, D. and Flavell, R. A. (1994). Local production of human IL-6 promotes insulitis but retards the onset of insulin-dependent diabetes mellitus in non-obese diabetic mice, *Int. Immunol.*, **6**, 1829–37.
[17] Wadt, K. A., Larsen, C. M., Andersen, H. U., Nielsen, K., Karlsen, A. E. and Mandrup-Poulsen, T. (1998). Ciliary neurotrophic factor potentiates the beta-cell inhibitory effect of IL-1beta in rat pancreatic islets associated with increased nitric oxide synthesis and increased expression of inducible nitric oxide synthase, *Diabetes*, **47**, 1602–8.
[18] Efrat, S., Linde, S., Kofod, H., Spector, D., Delannoy, M., Grant, S., Hanahan, D. and Baekkeskov, S. (1988). Beta-cell lines derived from transgenic mice expressing a hybrid insulin gene-oncogene, *Proc. Natl. Acad. Sci. USA*, **85**, 9037–41.
[19] Hamaguchi, K., Gaskins, H. R. and Leiter, E. H. (1991). NIT-1, a pancreatic beta-cell line established from a transgenic NOD/Lt mouse, *Diabetes*, **40**, 842–9.

[20] Kay, T. W., Parker, J. L., Stephens, L. A., Thomas, H. E. and Allison, J. (1996). RIP-beta 2-microglobulin transgene expression restores insulitis, but not diabetes, in beta 2-microglobulin null nonobese diabetic mice, *J. Immunol.*, **157**, 3688–93.

[21] Hilton, D. J., Hilton, A. A., Raicevic, A., Rakar, S., Harrison-Smith, M., Gough, N. M., Begley, C. G., Metcalf, D., Nicola, N. A. and Willson, T. A. (1994). Cloning of a murine IL-11 receptor alpha-chain; requirement for gp130 for high affinity binding and signal transduction, *EMBO J.*, **13**, 4765–75.

[22] Altschul, S. F., Gish, W., Miller, W., Myers, E. W. and Lipman, D. J. (1990). Basic local alignment search tool, *J. Mol. Biol.*, **215**, 403–10.

[23] Ausubel, F. M., Brent, R., Kingston, R. E., Moore, D. D., Seidman, J. G., Smith, J. A. and Struhl, K. (1994). *Current Protocols in Molecular Biology*, John Wiley and Sons, Inc., New York.

[24] Hilton, D. J. and Nicola, N. A. (1992). Kinetic analyses of the binding of leukemia inhibitory factor to receptor on cells and membranes and in detergent solution, *J. Biol. Chem.*, **267**, 10238–47.

[25] Schreiber, E., Matthias, P., Muller, M. M. and Schaffner, W. (1989). Rapid detection of octamer binding proteins with 'mini-extracts', prepared from a small number of cells, *Nucleic Acids Res.*, **17**, 6419.

[26] Wagner, B. J., Hayes, T. E., Hoban, C. J. and Cochran, B. H. (1990). The SIF binding element confers sis/PDGF inducibility onto the c-fos promoter, *EMBO J.*, **9**, 4477–84.

[27] Campbell, I. L. and Harrison, L. C. (1989). Viruses and cytokines: evidence for multiple roles in pancreatic beta cell destruction in type 1 insulin-dependent diabetes mellitus, *J. Cell. Biochem.*, **40**, 57–66.

[28] Campbell, I. L., Cutri, A., Wilson, A. and Harrison, L. C. (1989). Evidence for IL-6 production by and effects on the pancreatic beta-cell, *J. Immunol.*, **143**, 1188–91.

[29] Southern, C., Schulster, D. and Green, I. C. (1990). Inhibition of insulin secretion from rat islets of Langerhans by interleukin-6. An effect distinct from that of interleukin-1, *Biochem. J.*, **272**, 243–5.

[30] Gearing, D. P., Comeau, M. R., Friend, D. J., Gimpel, S. D., Thut, C. J., McGourty, J., Brasher, K. K., King, J. A., Gillis, S., Mosley, B. *et al.* (1992). The IL-6 signal transducer, gp130: an oncostatin M receptor and affinity converter for the LIF receptor, *Science*, **255**, 1434–7.

[31] Boulton, T. G., Stahl, N. and Yancopoulos, G. D. (1994). Ciliary neurotrophic factor/leukemia inhibitory factor/interleukin 6/oncostatin M family of cytokines induces tyrosine phosphorylation of a common set of proteins overlapping those induced by other cytokines and growth factors, *J. Biol. Chem.*, **269**, 11648–55.

[32] Stahl, N., Boulton, T. G., Farruggella, T., Ip, N. Y., Davis, S., Witthuhn, B. A., Quelle, F. W., Silvennoinen, O., Barbieri, G., Pellegrini, S. *et al.* (1994). Association and activation of Jak-Tyk kinases by CNTF-LIF-OSM-IL-6 beta receptor components, *Science*, **263**, 92–5.

[33] Vara, E., Arias-Diaz, J., Garcia, C. and Balibrea, J. L. (1994). Cytokine-induced inhibition of lipid synthesis and hormone secretion by isolated human islets, *Pancreas*, **9**, 316–23.

[34] Sandler, S., Bendtzen, K., Eizirik, D. L. and Welsh, M. (1990). Interleukin-6 affects insulin secretion and glucose metabolism of rat pancreatic islets *in vitro*, *Endocrinology*, **126**, 1288–94.

[35] Sjoholm, A. (1991). Cytokines inhibit proliferation and insulin secretion by clonal rat insulinoma cells (RINm5F) non-synergistically and in a pertussis toxin-insensitive manner, *Immunol. Lett.*, **30**, 81–6.

[36] Sjoholm, A. (1992). Differential effects of cytokines on long-term mitogenic and secretory responses of fetal rat pancreatic beta-cells [published erratum appears in *Am. J. Physiol.*, 1993 Apr;264(4 Pt 1):preceding C761], *Am. J. Physiol.*, **263**, C114–20.

[37] Schmidli, R. S., Faulkner-Jones, B. E., Harrison, L. C., James, R. F. and DeAizpurua, H. J. (1996). Cytokine regulation of glutamate decarboxylase biosynthesis in isolated rat islets of Langerhans, *Biochem. J.*, **317**, 713–9.

